# Transcranial Stimulation Methods in the Treatment of MDD Patients—The Role of the Neurotrophin System

**DOI:** 10.3390/ijms262411878

**Published:** 2025-12-09

**Authors:** Dragica Selakovic, Marina Mitrovic, Biljana Ljujic, Vladimir Janjic, Dragan Milovanovic, Nemanja Jovicic, Bojana Simovic Markovic, Irfan Corovic, Milica Vasiljevic, Pavle Milanovic, Momir Stevanovic, Sara Rosic, Suzana Randjelovic, Ermin Fetahovic, Anshu Chopra, Jovan Milosavljevic, Gvozden Rosic

**Affiliations:** 1 Department of Physiology, Faculty of Medical Sciences, University of Kragujevac, 34000 Kragujevac, Serbia; rosicsara@gmail.com (S.R.); anshchopra2904@gmail.com (A.C.); jovan.milosavljevic1997@gmail.com (J.M.); grosic@fmn.kg.ac.rs (G.R.); 2 Department of Medical Biochemistry, Faculty of Medical Sciences, University of Kragujevac, 34000 Kragujevac, Serbia; mitrovicmarina34@gmail.com; 3 Center for Molecular Medicine and Stem Cell Research, Faculty of Medical Sciences, University of Kragujevac, 34000 Kragujevac, Serbia; bojana.simovic@gmail.com (B.S.M.); ira.corovic@gmail.com (I.C.); 4 Department of Genetics, Faculty of Medical Sciences, University of Kragujevac, 34000 Kragujevac, Serbia; bljujic74@gmail.com; 5 Department of Communication Skills, Ethics, and Psychology, Faculty of Medical Sciences, University of Kragujevac, 34000 Kragujevac, Serbia; vladadok@yahoo.com (V.J.); erminfetahovic96@gmail.com (E.F.); 6 Department of Psychiatry, Faculty of Medical Sciences, University of Kragujevac, 34000 Kragujevac, Serbia; 7 Psychiatric Clinic, University Clinical Center Kragujevac, 34000 Kragujevac, Serbia; 8 Department of Pharmacology and Toxicology, Faculty of Medical Sciences, University of Kragujevac, 34000 Kragujevac, Serbia; piki@fmn.kg.ac.rs; 9 Department of Histology and Embryology, Faculty of Medical Sciences, University of Kragujevac, 34000 Kragujevac, Serbia; nemanjajovicic.kg@gmail.com; 10 Department of Dentistry, Faculty of Medical Sciences, University of Kragujevac, 34000 Kragujevac, Serbia; milicavaska13@gmail.com (M.V.); pavle11@yahoo.com (P.M.); momirstevanovic7@gmail.com (M.S.); 11 Department of Emergency Medicine, University Clinical Center Kragujevac, 34000 Kragujevac, Serbia; suzanarandjelovic25@gmail.com

**Keywords:** transcranial stimulation, depression, neurotrophins

## Abstract

Major depressive disorder (MDD) continues to be a primary cause of disability globally, with a significant number of patients exhibiting resistance to standard pharmacological and psychotherapeutic interventions. In recent years, non-invasive brain stimulation techniques, especially transcranial magnetic stimulation (TMS) and transcranial direct current stimulation (tDCS), have emerged as promising therapies for treatment-resistant MDD. A comprehensive search was performed in PubMed, which included all studies published over the last ten years. Eligible studies encompassed both animal models and clinical investigations. This review provides a comparative overview of transcranial electrical stimulation modalities, with a focus on their mechanisms of action, clinical efficacy, and underlying neurobiological mechanisms. We pay particular attention to the role of the neurotrophin system, specifically brain-derived neurotrophic factor (BDNF), in mediating the treatment effects of transcranial stimulation. Recent findings indicate that neuromodulation could improve neuroplasticity by increasing BDNF levels and associated signaling pathways, which may help stabilize mood and enhance the improvement of individuals with MDD. A more profound understanding of these mechanisms could lead to more precise, biomarker-driven interventions. Further research is essential to elucidating the long-term effects of brain stimulation on neurotrophin levels and to creating more individualized treatment strategies.

## 1. Introduction

Major Depressive Disorder (MDD) remains one of the most frequently occurring psychiatric disorders, which can severely affect the patient’s life quality [1]. It is typically characterized by a loss of interest in daily activities (anhedonia), chronic feelings of sadness, and various somatic and cognitive issues. MDD represents a major global public health issue that is marked by substantial suffering, considerable difficulties in daily life, and frequently poor responses to conventional treatments [2].

### 1.1. Transcranial Stimulation Methods in the Treatment of MDD Patients

Current psychiatric guidance for MDD treatment is based on the pharmacological approach (antidepressants) and cognitive-behavioral psychotherapy as the first-line therapy. While there is a noticeable improvement in their effectiveness, their use is often accompanied by side effects and may not be accessible to certain groups of patients. Given the significant health burden of MDD, it is essential to investigate additional treatment protocols. The ongoing demand for alternative therapeutic approaches to MDD is driven by the fact that nearly one-third of MDD patients exhibit pharmacotherapy resistance to conventional antidepressants (in a timely manner), and transcranial stimulation methods may be considered beneficial for those patients. However, despite the growing acceptance of transcranial stimulation methods, largely due to positive results from clinical trials, most of the available data still support the notion that these methods achieve therapeutic success primarily when used in conjunction with conventional antidepressants. Despite significant limitations to transcranial stimulation techniques, such as stigma, cognitive side effects, the potential need for anesthesia, daily sessions over several weeks, and slower response rates [3], these methods still provide a reasonable and reliable alternative for patients with MDD, especially when considering the impact of undereducated professionals, patients, and the public. However, a more profound understanding of the neurochemical changes associated with these therapeutic approaches is essential to address skepticism and establish them as a reliable treatment protocol.

Transcranial stimulation techniques employed as adjunctive therapy for individuals with MDD can be categorized into two principal types: non-invasive and invasive [4]. This classification is based on several criteria, including the necessity for anesthesia or analgesics, the direct surgical placement of electrodes in specific brain regions, and the maintenance of skin integrity. However, there is no total consensus among regulatory authorities due to variations in national guidelines and differing professional attitudes. As presented in Table 1, non-invasive techniques for treating MDD include transcranial magnetic stimulation (TMS), transcranial direct current stimulation (tDCS) and alternating current stimulation, transcranial random noise stimulation (tRNS), transcranial photobiomodulation (tPBM), transcranial ultrasound stimulation (TUS) and focused ultrasound stimulation (FUS), trigeminal nerve stimulation (TNS), and electroconvulsive therapy (ECT). In contrast, invasive techniques consist of deep brain stimulation (DBS), vagus nerve stimulation (VNS), and epidural cortical stimulation.

**Figure 1:**
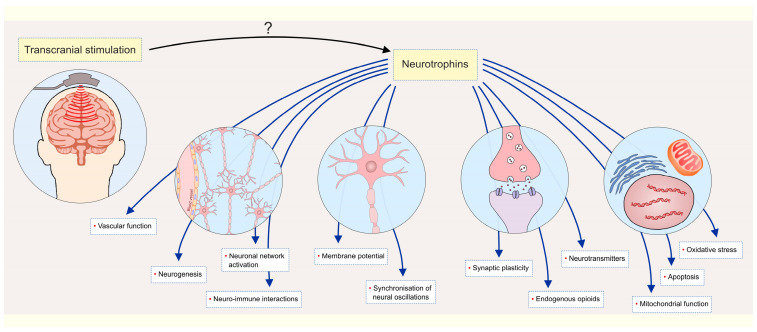
An overview of the neurotrophin system’s effects on brain tissue in response to transcranial stimulation.

Many of those beneficial mechanisms are also addressed as being regulated by the neurotrophin system (Figure 1). For this purpose, we provide an overview of literature data from the past decade that emphasizes the effects of transcranial stimulation-induced alterations on neurotrophin system elements related to depression. We propose that these alterations may serve as potential biomarkers for assessing the benefits of transcranial stimulation in the treatment of MDD.

### 1.2. Neurotrophin System and MDD

MDD is highly complex and influenced, among other things, by genetic susceptibility, environmental factors, and alterations in the brain’s chemistry [1]. Recent research on the neurotrophin system and MDD has gained momentum, with particular emphasis on brain-derived neurotrophic factor (BDNF) and, to a lesser extent, neurotrophin-3 (NT-3) and nerve growth factor (NGF) as key players [5,6,7]. These neurotrophins are crucial for neurons in various aspects, including survival, growth, differentiation, and especially synaptic plasticity, which serves as the primary mechanism of communication between brain circuits. Consequently, malfunction in this process is one of the major reasons for the development of MDD [8,9]. As a result, the neurotrophin system plays a major role in the pathophysiology of MDD through various mechanisms, mainly impacting the areas of neuroplasticity, neurogenesis, and neurotransmitter modulation [10]. Therefore, it is important to understand the complex relationship between the neurotrophin system and MDD. This knowledge can provide valuable perspectives on the disorder’s pathophysiology and create avenues for the development of novel therapeutic approaches.

The neurotrophin system comprises a family of secreted proteins that exert their effects by binding to specific transmembrane receptors, primarily the Trk family (TrkA for NGF, TrkB for BDNF and NT-4/5, and TrkC for NT-3) and the p75 neurotrophin receptor (p75NTR), which can bind to all neurotrophins. Neurotrophins bind to the Trk receptor and activate downstream signaling cascades, including the PI3K/Akt pathway (promoting survival), the MAPK/ERK pathway (promoting growth and differentiation) and the PLCγ pathway (involved in synaptic plasticity and neurotransmitter release), while their binding to p75NTR, although it can initiate survival, mainly activates cell death pathways, depending on the co-receptor context and the specific neurotrophin [11,12]. Specifically, neurotrophin BDNF has emerged as an important player in the pathogenesis of MDD and a potential diagnostic biomarker for this disorder [13]. It is distributed across the brain, but it is highly expressed in the hippocampus, amygdala, and prefrontal cortex. These areas are directly involved in mood regulation, learning, memory, and stress response, all of which have been shown to be affected by MDD [14]. BDNF is essential factor for neurogenesis and synaptogenesis, and both processes are important for keeping brain circuits healthy and flexible. Additionally, BDNF promotes synaptic plasticity, the capacity of synapses to be enhanced or diminished over time, which constitutes the foundation of learning and memory and is thought to be compromised in MDD [15].

An increasing amount of evidence gathered from both human studies and animal models indicates a major imbalance in the neurotrophin system in MDD. One of the clearest and most compelling findings in the pathophysiology of MDD is the low BDNF levels [16]. Multiple meta-analyses have produced consistent findings that serum BDNF concentrations are lower in depressed patients than in healthy non-depressed controls. The decrease in BDNF is in most cases related to the extent of the depressive symptoms, which suggests a strong connection between the deficiency in BDNF and the clinical outcome of MDD. Furthermore, the studies have also suggested that blood BDNF levels could increase following post-antidepressant medication in MDD, indicating its supportive role [17,18]. Contrary to most prior findings, data obtained in the most recent nested case–control study conducted by Wang and colleagues [19] showed elevated plasma BDNF levels in a cohort of 52 middle-aged women (mean age = 56) who later developed MDD. This elevation in BDNF levels was particularly pronounced in MDD cases diagnosed two to five years after the baseline assessment. The authors propose that BDNF may serve as a potential risk biomarker for the onset of MDD, challenging its previously recognized protective function.

Additionally, various studies have examined serum levels of BDNF and its proBDNF form, finding that BDNF levels decreased while proBDNF levels increased in patients with MDD. Accordingly, Gelle and the team [20] specifically demonstrated that BDNF and proBDNF levels changed inversely. They found that the BDNF/proBDNF ratio significantly increased in both serum and exosomes among 42 patients with MDD who were treated with antidepressant therapy, which included either an antidepressant drug or repeated transcranial magnetic stimulation (rTMS) over a period of 3 to 7 weeks. However, the authors found no relationship between BDNF and proBDNF levels, clinical improvement (cognitive complaints), and depression scales (depression intensity).

In a separate study, Yang and collaborators [21] examined the relationship between the tissue plasminogen activator (tPA)/plasminogen activator inhibitor-1 (PAI-1) system, the BDNF/proBDNF ratio, and SSRI antidepressant treatment over a 4-week period in 57 patients with MDD. The authors showed that patients with MDD had significantly decreased levels of BDNF and tPA, as well as markedly increased levels of proBDNF and PAI-1 in their peripheral blood before SSRI treatment; they also discovered that the tPA/PAI-1 ratio and BDNF/proBDNF ratio normalized after SSRI treatment. Furthermore, they established that the combination of tPA, PAI-1, and BDNF exhibited the most significant diagnostic value for MDD, indicating that their interaction may be crucial in MDD and its treatment. In line with this, another study highlighted that patients experiencing their first episode of drug-free MDD and responding to SSRI treatments exhibited elevated serum BDNF levels [22]. In general, various researchers found that patients with untreated MDD have lower blood BDNF levels than healthy controls and that the antidepressant treatments increased blood BDNF levels more significantly after pharmacological treatment compared to non-pharmacological therapy [23]. Moreover, Zwolińska and her team [24] found that proBDNF levels decreased considerably with antidepressant therapy in 31 female patients diagnosed with MDD, while BDNF levels did not change significantly. They suggested that proBDNF may be used as a biomarker for recovery from depression and a marker of mood improvement. Furthermore, in the preclinical study investigating the role of peripheral proBDNF in triggering depression-like behaviors in mice, Lin and coworkers [25] demonstrated that C57BL/6 mice injected with AAV-proBDNF developed depression-like behaviors four weeks post-injection, which lasted for 12 weeks. These behaviors were characterized by increased immobility time in both the tail suspension test and forced swim test, reduced sucrose consumption, and a decrease in the density and length of dendritic spines in both the dentate gyrus and amygdala; all these effects were reversed by injecting an anti-proBDNF antibody. These results suggest proBDNF’s potential as a pathogenic factor in depression.

Chronic stress has been recognized as a primary risk factor for significant MDD, and its adverse consequences have been, in part, through the dysregulation of the neurotrophin system. Stress, especially during crucial developmental phases or extended exposure in adulthood, can induce a series of neurobiological alterations, encompassing modifications in the hypothalamic–pituitary–adrenal (HPA) axis, neuroinflammation, and diminished neuroplasticity. A study performed by Philpotts and associates [26] showed that chronic stress was associated with reduced hippocampal BDNF levels, which are crucial for neuroplasticity and mood regulation, contributing to a depressive-like phenotype in MDD. Furthermore, the authors demonstrated that incorporating Crocus sativus (saffron) into the diet can boost hippocampal BDNF levels and alleviate depression symptoms. In another study that employed a CUMS-induced mouse animal model of depression, Yang and collaborators [27] demonstrated that chronic stress significantly elevated proBDNF, p75NTR, and sortilin levels, while decreasing mature BDNF/TrkB levels in the cortex and hippocampus of mice, providing evidence that there is an imbalance between proBDNF/p75NTR/sortilin and BDNF/TrkB in the pathogenesis of depression, and it can, to some extent, be reversed by antidepressant treatment.

Long-term stress might increase the levels of proBDNF, which can alter the BDNF signaling pathway and make depression worse by triggering the inflammatory reactions in the immune cells. The research led by Yang et al. [28] focused on proBDNF/p75NTR signaling in immune cells and investigated its relationship with the inflammatory markers in patients with MDD. They demonstrated that proBDNF/p75NTR signaling is upregulated in the immune cells of MDD patients, particularly in CD4+ and CD8+ T cells, and plays a role in modulating inflammatory responses; increases in IL-1β and IL-10 levels were positively correlated with major depression scores. Interestingly, sortilin levels were found to be positively linked with IL-1β. The authors propose that proBDNF/p75NTR signaling might not only be used as biomarker but also as a treatment target for MDD. Contrary to previous research, Li and the associates [29] observed increased BDNF levels in MDD patients (*n* = 83) with higher suicidal ideation that positively correlated with the sum score of the Beck Scale for Suicide Ideation (BSS), as well as a significant elevation of tPA, IL-1β, and IL-6 levels. Patients suffering from MDD, on the other hand, had reduced TrkB and proBDNF levels. The authors put out the concept that BDNF is involved in the neurobiology of the link between MDD and suicidal thoughts, specifically in the way BDNF-TrkB signaling interacts with inflammatory variables, MDD clinical features, and other factors.

Furthermore, multiple studies have revealed that high glucocorticoid levels, such as corticosterone, a hallmark feature of chronic stress and HPA axis dysregulation in MDD, can reduce BDNF production, inhibiting adult hippocampal neurogenesis in depression [30]. In line with this, Herhaus and his team [31] examined the correlation between BDNF and cortisol in response to stress, specifically employing the Trier Social Stress Test (TSST) on 29 healthy young males. The research concluded that exposure to acute stress significantly increased both BDNF and cortisol levels. A more pronounced cortisol response was associated with the quicker decline of BDNF after stress. Moreover, the exposure to chronic stress was associated with lower basal BDNF levels. While BDNF may have a neuroprotective function after acute stress, the authors speculated that chronic stress’s elevation in cortisol levels would impede BDNF recovery and raise the risk of neurodegeneration. Numerous studies have revealed that one of the most effective therapies for enhancing neuroplasticity and resilience is to restore BDNF expression, which is lowered by chronic stress, including aerobic exercise and various pharmacological therapies (TrkB agonists; antidepressant drugs such as ketamine; AAV-BDNF and mRNA-BDNF overexpression [32,33,34]).

Genetic differences in the neurotrophin genes and their receptors have been proposed to shape the predisposition to developing MDD. Genetic variations in the brain-derived neurotrophic factor (BDNF) gene, particularly the Val66Met (rs6265) variant, have been found to alter BDNF secretion and processing and to be linked with higher risk of MDD as well as weaker efficacy of antidepressant therapy [35]. However, a further investigation conducted by Wang and colleagues [36] found no substantial correlation between the BDNF Val66Met variant and MDD, which is contrary to the previous results. Consequently, despite extensive research on the BDNF gene’s Val66Met polymorphism, a number of results regarding its correlation with MDD remain inconclusive [37,38].

The dysregulation of BDNF levels in MDD has a significant impact on the anatomy and physiology of the brain regions, including the hippocampus, the prefrontal cortex (PFC), and the amygdala, which are mainly responsible for mood control, emotional expression, and cognitive activities [39]. The hippocampus is a critical center that supports learning, memory, and emotional regulation, and it is typically accompanied by the loss of volume and a decrease in neurogenesis in the case of MDD. The reduced levels of BDNF in the hippocampus are the key factor contributing to these changes, because BDNF is essential for promoting neurogenesis and synaptic plasticity in this region. In contrast, proBDNF is thought to act as an inhibitor [40]. Depressed patients are likely to suffer from disturbances in brain circuits involved in mood regulation and cognitive functions, resulting in mood disorders such as MDD. Long-term potentiation (LTP) and long-term depression (LTD) influence cognitive and affective processes. Increased neuronal firing enhances LTP, while neuronal activity reduces long-term depression (LTD). The BDNF-TrkB cellular pathway modulates the late phase of LTP through activation of different signaling cascades (PI3K/Akt/CREB, MAPK/ERK, and mTOR) that are responsible for the increase in glutamate sensitivity and the enlargement of dendritic spines, particularly in the hippocampus [41].

Moreover, the research conducted by Lee and collaborators [42] indicated that elevated serum BDNF levels are positively associated with greater resting-state functional connectivity (RSFC) between the hippocampus and prefrontal areas, implying improved cognitive performance in adolescents with MDD. This connectivity correlates with reduced attention issues, suggesting that BDNF may enhance cognitive functions by modulating hippocampal-prefrontal interactions. According to this, Wan and colleagues [43] in a different study discovered that repeated ketamine administration in mice during the neonatal stage produces cognitive impairments (as measured using the Morris water maze and the novel object recognition test (NORT) in adulthood) due to the damage to synaptic plasticity and excitability. The authors considered the implications of BDNF in these deficits and found that the cognitive decline is linked to the lowered levels of BDNF, the damage to synaptic plasticity, and the diminished excitability of the glutamatergic neurons in the CA1 area of the hippocampus. Injecting pAdeno-CaMKIIα-BDNF-mNeuronGreen into the hippocampus CA1 region of mice causes BDNF to be overexpressed, subsequently causing the mice’s synaptic plasticity and cognitive performance to improve.

The prefrontal cortex (PFC) is vital for controlling emotions, making decisions, and other executive processes. MDD is often characterized by hypoactivity and structural changes in the PFC [44]. Lower levels of BDNF in the PFC are linked to impairments with synaptic plasticity, lower neuronal excitability, and declines in cognitive control. All of these complications can lead to anhedonia, apathy, and problems with executive function in MDD patients [45]. Since BDNF positively affects synaptic plasticity and neuronal excitability in the PFC, its deficit in MDD has been linked with a shrinkage of gray matter and a change in functional connectivity among the PFC circuits. The reduced availability of BDNF can interfere with the PFC’s role of regulating the amygdala, which is responsible for the processing of fear, thus resulting in the latter’s overactivity, thereby evoking and amplifying negative emotions [46].

The amygdala is particularly important in the processing of emotions related to fear and danger. Dysregulation of the amygdala, often characterized by hyperactivity, is a hallmark of MDD [47]. The direct effects of neurotrophins on amygdala hyperactivity in MDD are currently under investigation; nevertheless, alterations in BDNF signaling within the amygdala may modify its excitability and lead to heightened negative affect and anxiety symptoms [48]. The stimulation of the CREB/BDNF pathway in the amygdala subsequent to psychological stress has been associated with the overexpression of synaptic GluA1 through mTOR signaling, thus disrupting the synaptic plasticity of the amygdala and ultimately promoting depression [49].

Although studies of BDNF’s role in MDD have been more prevalent, alterations in other neurotrophins also play roles in MDD, with results that have been inconsistent. For instance, Salsabil and associates [50] demonstrated increased serum NGF levels in MDD patients, potentially due to higher inflammation or activation of the stress pathway. However, a separate study performed by Maes and his team has revealed no difference or even lower NGF levels in MDD, indicating the complexity of NGF’s role in this depressive disorder [51]. Research on other neurotrophins’ roles in MDD, such as NT-3, is less extensive compared to BDNF. Some previous studies reported reduced NT-3 expression and decreased BDNF in post-mortem brain tissue and peripheral blood cells of patients with MDD [52]. These findings suggested that NT-3 could be a potential novel pharmaceutical target for MDD due to its effects on the HPA axis, synaptic plasticity and neurogenesis regulation, BDNF signaling stimulation, and monoamine neurotransmitters [53]. Interestingly, the recent study performed by Kasakura and colleagues [54] proposed that NT-3 downregulation induced by chronic antidepressant treatment may serve as a negative feedback mechanism to prevent excessive increases in neurogenesis and the key factor associated with both adult neurogenesis and emotional regulation, NPY expression, thereby maintaining homeostasis.

The growing body of evidence points to a disturbance in the neurotrophin signaling system, which is marked by a decrease in the levels of BDNF in the main areas of the brain, such as the hippocampus and the prefrontal cortex, leading to the impairment of both the structure and function of the brain that are related to the symptoms of MDD. Chronic stress has a significant effect on this system, exacerbating neurotrophin deficiency. Comprehending this complex interaction will allow the researchers to clarify the aspects of MDD pathogenesis and to facilitate the development of innovative therapeutic approaches aimed at neurotrophic pathways, including the use of transcranial stimulation methods to treat patients with MDD.

## 2. Methods

**Figure 2:**
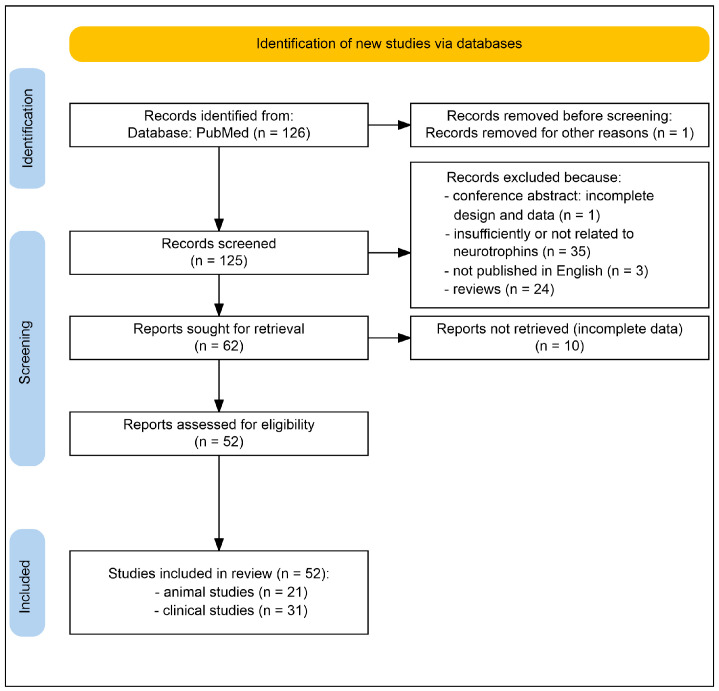
The flow diagram shows the process of screening and selecting studies.

This review was conducted following the guidelines of the Preferred Reporting Items for Scoping Reviews and Meta-Analyses (PRISMA) [55]. A comprehensive search was performed in PubMed, which included all studies published over the last ten years until October 2025 (Figure 2). The search strategy utilized Boolean operators to combine the following terms: “neurotrophin” AND “depression” AND (“transcranial magnetic stimulation” OR “transcranial current stimulation” OR “electroconvulsive therapy” OR “transcranial photobiomodulation” OR “transcranial ultrasound stimulation” OR “transcranial random noise stimulation” OR “deep brain stimulation” OR “vagus nerve stimulation”). Additional records were identified by screening the reference lists of the included papers and relevant reviews. Eligible studies encompassed both animal models and clinical investigations that directly focused on the topic. Inclusion criteria required a detailed description of the transcranial therapeutic strategies associated with depression, as well as the mechanistic role of elements within the neurotrophin system. The exclusion criteria included review articles, non-peer-reviewed papers, conference abstracts, papers that did not address the topic, case reports lacking experimental data, and articles unavailable in the English language. Two reviewers conducted an independent screening of titles and abstracts, subsequently retrieving full texts for studies that appeared to be potentially relevant. Interrater reliability was high (Cohen’s kappa = 0.9674 ± 0.089). The two reviewers independently recorded the data, engaged in discussions about the findings, and regularly revised the data through an iterative approach. Disagreements were resolved through consensus among the co-authors.

This review is limited by the fact that the literature search was conducted solely on PubMed, as other databases were not accessible. In addition, the number of presented invasive techniques was limited. Heterogeneity of the obtained results appears due to numerous factors: A—stimulation parameters and design for various applied techniques; B—sample characteristics, including sample size, age, gender, pretreatment, comorbidity, and other therapeutic protocols employed in clinical trials; C—species characteristics, methodology for depression induction, age, and previous genetic interventions in animal research; D—assay methods.

## 3. Results and Discussion

### 3.1. An Overview of Non-Invasive Methods of Transcranial Stimulation Impact on the Neurotrophin System Elements in the Treatment of Depression

As previously mentioned, non-invasive methods of transcranial stimulation in the treatment of MDD are more frequently reported in clinical trials, but also in preclinical experimental models exclusively performed in rodents. However, as shown in Table 2, Table 3, Table 4 and Table 5, the incidence of the applied methodologies, as well as the parameters estimated in the evaluation of non-invasive methodologies’ impact on the neurotrophin system elements in the treatment of depression, is not proportional and does not allow full insight into this relationship.

#### 3.1.1. Transcranial Magnetic Stimulation

The antidepressant potential of transcranial magnetic stimulation (TMS) has been employed in clinical practice for thirty years. Initiated by reports of hypofunction of the left prefrontal cortex in depression, the investigator team from the National Institute of Mental Health in Charleston, led by Mark George [56], used rapid-rate TMS to activate neurons near the brain surface in six patients with severe medication-resistant depression, two of whom showed robust mood improvement. Shortly after, Pascual-Leone and coworkers [57] conducted the first clinical trial involving a sufficient number of medication-resistant depression patients, which confirmed the benefits of rTMS as a safe, non-convulsive method for MDD patients. Over the past three decades, numerous clinical trials, as well as studies conducted in animal experimental models, have offered plentiful evidence for rTMS effects, especially in treating treatment-resistant MDD, which has been identified as the principal clinical indication for the use of rTMS. Numerous biological mechanisms have been implicated in the antidepressant effects of rTMS, including the neurotrophin system. However, there is still no consensus considering the impact of neurotrophin system elements on the clinical response to rTMS.

Liu and colleagues [58] performed a clinical trial on 130 depressive patients (depressive disorder with non-suicidal self-injury behavior) to evaluate the potential of rTMS in conjunction with sertraline and to compare it with the group of patients treated by sertraline alone (the initial dose of 25 mg/day, which was gradually adjusted to 50 mg/day, orally, once a day). The rTMS protocol, which used a frequency of 10 Hz and an intensity of 80% of the resting exercise threshold, employed a cyclic treatment method, with each session lasting 5 s and then a 15-s rest interval, for a total of 15 min per day. This protocol was administered for 5 days each week, with a 2-day break, over the course of 4 weeks, mirroring the schedule used for the sertraline-only group. The repeatable battery for the assessment of neuropsychological status showed that the antidepressant effect in the combined group was stronger when compared to the sertraline-alone group (the total clinical effective rate of 95.38% vs. 84.61%). The evaluation of serum samples revealed that the levels of BDNF and NGF were higher in the combination group compared to the single treatment groups (Table 2A), which was accompanied by a reduction in neuroinflammatory markers and an increase in estimated neurotransmitters (norepinephrine, dopamine, and 5-hydroxytryptamine), thereby confirming the efficacy of rTMS in enhancing antidepressant function.

**Table 2:** (A). An overview of transcranial magnetic stimulation methods’ impact on the neurotrophin system in MDD patients. (B). An overview of transcranial magnetic stimulation methods’ impact on the neurotrophin system in depression-induced animal models.

**(A)**							
**Applied Methodology**	**Diagnosis**		**Neurotrophin System Alterations**			**Clinical Outcome**	**Ref.**
	**Type**	**Specific Features**	**Neurotrophins**	**Neurotrophin Receptors**	**Downstream Mechanisms**		
rTMS (10 Hz, intensity 80% of the resting exercise threshold, a cyclic treatment approach, duration—5 s with an interval of 15 s, the total treatment time—15 min, once per day) 5 days a week, for 4 weeks)	MDD (*n* = 130)	Non-suicidal self-injury behavior + sertraline	BDNF ↑ NGF ↑	-	Norepinephrine ↑ Dopamine ↑ 5-hydroxytryptamine ↑ Neuroinflammation ↓	Positive	[58]
rTMS (10 Hz, total of 37.5 min, 4 s of stimulation time and 26 s of latent time, 20 sessions, every weekday for a month)	MDD (*n* = 51)	-	BDNF ↑ GDNF ↑	-	-	Positive	[59]
rTMS (10 Hz for 3 weeks—5 sessions per week)	MDD (*n* = 6)	Geriatric depression	BDNF n.c. NGF n.c.	-	-	Initial—positive Follow up—n.c.	[60]
rTMS (10 Hz, intensity of 120% motor threshold, 25 trains of 8 s, and an inter-train interval of 26 s, 5 days per week, for 4 weeks)	Mild to moderate DD (*n* = 100)	+ agomelatine	BDNF ↑	-	Norepinephrine ↑	Positive	[61]
rTMS (10 Hz, the motor threshold was 80% to 110%, stimulation for 5 s, interval of 25 s, once a day for 20 min, 5 times per week, for 8 weeks)	MDD (*n* = 120)	Middle-aged and elderly + escitalopram	BDNF ↑	-	-	Positive	[62]
rTMS (20 Hz, duration of 2 s, 20 times at 30 s intervals, 100% of the motor threshold, 5 days a week for 4 weeks)	MDD (*n* = 66)	Treatment-resistant depression	BDNF ↑ GDNF ↑	-	-	Positive	[63]
rTMS (iTBS and cTBS), for 2 weeks	MDD (*n* = 48)	Treatment-resistant depression	BDNF ↑ NGF n.c.	-	-	Positive	[64]
rTMS (iTBS), for 3–6 weeks	MDD (*n* = 25)	Treatment-resistant depression	BDNF ↑	-	-		[65]
LFMS (20 min per session, 5 sessions per week, for 6 weeks): RAS and RDS	MDD (*n* = 29)	-	BDNF ↑	-	-	Positive	[66]
neuronavigation-guided rTMS (10 Hz, 5 s duration, 15 s inter-train intervals, 6000 pulses per session, 100% of the MT) for 7 days (120 trains)	MDD (*n* = 59)	Treatment-naive depressive patients with suicidal ideation	BDNF ↑	TrkB ↓	-	Positive	[67]
Arrows (↑ or ↓) indicate *p* < 0.05; n.c. indicates *p* > 0.05; *n* indicates the number of subjects.							
**(B)**							
rTMS (10-Hz for 3 weeks)	Depression in rats	CUMS	-	-	FGF2/FGFR1/p-ERK ↑	Positive	[68]
rTMS (15 and 25 Hz) for 4 weeks	Depression in mice	CUMS	BDNF ↑	TrkB ↑	p11/BDNF/Homer1a ↑	Positive	[69]
rTMS (10 Hz, 5 s per train, 20 trains per day) for 28 days	Depression in mice	CUMS + fluoxetine	BDNF ↑	TrkB ↑	BDNF/TrkB ↑	Positive	[70]
iTBS for 2 weeks: 1800 pulses, 720 s 2400 pulses, 960 s 3000 pulses, 1200 s	Depression in rats	Poststroke + CUMS	BDNF ↑	-	cAMP/PKA/CREB ↑	Positive	[71]
rTMS (10 Hz, 3 min) for 4 weeks: medium-intensity—50 mT high-intensity—1 T	Depression in mice	Olfactory bulbectomy	BDNF ↑	-	Cell proliferation ↑ Neurogenesis ↑	Positive	[72]
Arrows (↑ or ↓) indicate *p* < 0.05.							

In accordance with the results of the previous study, a randomized case–control study by Ozkan and coworkers conducted in MDD patients showed the benefits of rTMS [59]. The serum levels of neurotrophins, specifically BDNF and glial cell line-derived neurotrophic factor (GDNF), which were initially lower in the patient group compared to healthy subjects, significantly increased following the rTMS protocol (10 Hz, a total of 37.5 min, 4 s of stimulation time, and 26 s of latent time, 20 sessions every weekday for a month), as shown in Table 2A. The clinical benefits of rTMS were confirmed using the Montgomery–Asberg Depression Rating Scale (MADRS).

Nevertheless, a multimodal case series study performed by Nicoletti and collaborators [60] to explore the pleiotropic effects of rTMS in geriatric depression did not confirm an increase in rTMS-induced neurotrophins (BDNF and NGF) following the administered protocol (a total of 15 sessions of 10 Hz for three weeks, five sessions per week, with a 2-day interval), despite an initial improvement in mood. The authors attribute the discrepancy of their results, compared to many similar reports, to the overlapping effects of multiple pharmacotherapies and various cerebrovascular comorbidities.

The study presented by Pu and investigators showed that rTMS was beneficial for patients with mild to moderate DD [61]. In addition to the significant improvement in the associated sleep disorder, rTMS (10 Hz, intensity of 120% motor threshold, 25 trains of 8 s, and an inter-train interval of 26 s, for 5 successive days per week over 4 weeks), when applied simultaneously with agomelatine (daily dose ranging from 25 to 75 mg based on clinical response and side effects for 8 weeks), led to a reduction in depression as measured by the Hamilton Depression Rating Scale (HDRS). The clinical outcome was accompanied by increased serum levels of BDNF and norepinephrine (Table 2A). Notably, the applied therapeutic approach showed benefits that were obvious from the very beginning of the trial and gradually increased in the following weeks, reaching the maximum after two months.

A similar experimental design was used in the clinical trial conducted by Wang and colleagues, focusing on middle-aged and elderly patients with MDD [62]. The authors again applied rTMS (10 Hz, with the motor threshold set between 80% and 110%, stimulation lasting for 5 s, followed by a 25-s interval, administered once daily for 20 min, 5 days a week, over a duration of eight weeks) in combination with escitalopram (5–20 mg/d). Compared to oral escitalopram alone, the combination of rTMS and escitalopram demonstrated a more effective antidepressant effect, as indicated by clinical evaluations using HDRS and the Repeatable Battery for the Assessment of Neuropsychological Status. Additionally, this combined protocol resulted in increased serum BDNF levels, as shown in Table 2A.

Demiroz and colleagues employed the rTMS protocol in the treatment of resistant depression [63]. The authors concluded from the results of this trial that rTMS (20 Hz, 2-s duration, administered 20 times at 30-s intervals, at 100% of the motor threshold, five days a week for four weeks) produced an antidepressant effect, which was confirmed by the HDRS and the Clinical Global Impression Scale (CGI) and was associated with the normalization of serum BDNF and GDNF levels.

In the study conducted by Valiuliene and investigators [64], the efficiency of rTMS in treating MDD patients with treatment-resistant depression was compared to that of MDD patients who responded to medication. The experimental design included an rTMS protocol for treatment-resistant depression (TRD) patients, which involved intermittent theta burst (iTBS) and continuous theta burst (cTBS), along with the administration of antidepressants and their combinations as recommended, serving as the positive control group. Both protocols were carried out over a two-week period and demonstrated a significant antidepressant effect, as indicated by the results from the MADRS and HDRS, with a more pronounced effect with the pharmacological approach. BDNF levels significantly increased after two weeks of the rTMS protocol (Table 2A); however, there was no significant change in serum NGF levels. The significant finding in this trial was a strong correlation between BDNF serum levels and MADRS, supporting the hypothesis for the potential role of BDNF as a biomarker in the evaluation of MDD.

Previous research by Valiuliene and her team [65] also evaluated the impact of TMS in treatment-resistant MDD patients, but using only iTBS rTMS as a therapeutic protocol for 3 to 6 weeks (five times a week, depending on the clinical outcome). The iTBS rTMS protocol resulted in an antidepressant effect (confirmed by a significant decline in the MADRS and HAM-D scales) while significantly increasing BDNF serum levels. Interestingly, BDNF levels did not statistically differ between healthy controls and treatment-resistant MDD patients at baseline, but brain stimulation increased BDNF levels only in psychiatric patients.

Xiao and collaborators [66] presented results on the use of rhythmic low-field magnetic stimulation (LFMS) in patients with MDD. Researchers applied LFMS (20 min per session, 5 sessions per week, for 6 weeks) in the form of rhythmic alpha stimulation (RAS) or rhythmic delta stimulation (RDS). The clinical evaluation of the patients (using HDRS and CGI) showed that both protocols resulted in antidepressant effects, with a significantly higher effect size in the RDS group. At the same time, the increase in serum BDNF levels was more pronounced in the RAS group. Following the obtained results, the authors proposed that a low baseline serum BDNF level may be a predictive biomarker for the efficacy of rhythmic magnetic stimulation.

An advanced method for rTMS, specifically neuronavigation-guided rTMS, was utilized by Pan and investigators [67] to assess this therapy in treatment-naive depressive patients experiencing suicidal ideation. A structural MRI-guided method (10 Hz, 6000 pulses per session, 100% of the MT parameter for rTMS treatment) was employed for 7 consecutive days. The session consisted of 6000 stimuli per day, delivered in 120 trains of 5 s at 10 Hz, with inter-train intervals of 15 s. Both active (patients) and sham groups showed a decline in HAMD and MADRS, as well as the Beck Scale for Suicide Ideation (BSI); however, the change was significant only in the patients’ group. The authors observed that the levels of BDNF following the neuronavigation-guided rTMS protocol were elevated in the active group compared to the sham group. In contrast, TrkB levels in the active group decreased relative to their baseline measurements. Additionally, there was no significant correlation found between the changes in BDNF or TrkB levels and the clinical variables.

An obvious difference in the results obtained from the presented publications could be attributed to the lack of standardized methodology and patient selection. Additionally, the polymorphism of elements within the neurotrophin system may also play a role in the antidepressant response to rTMS [73,74], as it has been established that MDD patients with Met alleles had more benefits from the prolonged intermittent theta-burst stimulation intervention.

Although Yan and coworkers [68] did not evaluate any specific elements of the neurotrophin system, they analyzed the impact of repetitive transcranial magnetic stimulation (rTMS) on another neurogenesis-regulating growth factor, fibroblast growth factor 2 (FGF2). The experimental protocol consisted of a standard procedure for CUMS induction of depression in rats (5 weeks) and the application of rTMS (10 Hz for 3 weeks). Behavioral testing (sucrose preference, forced swimming, and open field test) indicated a decrease in CUMS-induced depressive-like behavior, along with an elevation of FGF2 expression in the hippocampus and prefrontal cortex. The authors suggested that the antidepressant effects of rTMS might be linked to the activation of the FGF2/FGFR1/p-ERK signaling pathway (Table 2B). Zuo and the investigators [69] provided more detailed insight into the impact of rTMS on CUMS-induced depressive-like behavior. Following the 8-week CUMS protocol, the authors employed higher frequencies for rTMS than in the previous study (15 Hz and 25 Hz) and administered them for a duration of 4 weeks. Behavioral testing, which included sucrose preference, open field, forced swimming, and tail suspension tests, revealed the antidepressant effects of rTMS at both frequencies, with 15 Hz proving to be more effective. Both frequencies of rTMS correlated with elevated expression of BDNF and TrkB in samples from PFC and hippocampus (Table 2B). Additionally, histological analyses validated neuroprotection by demonstrating the prevention of neuronal death, the stimulation of neurogenesis, and the facilitation of synaptic plasticity. The authors additionally suggested that the p11/BDNF/Homer1a signaling pathway may play a role in the neuroprotective benefits of rTMS.

To examine the effects of rTMS compared to the standard pharmacological approach on CUMS-induced depressive-like behavior in mice, Yuan and collaborators [70] performed the standard CUMS protocol and subsequently administered rTMS (10 Hz for 5 s per train, a total of 20 trains per day) and/or fluoxetine (5 mg/kg/day, intraperitoneally) for a duration of 28 days. An extensive battery of behavioral tests revealed the antidepressant effects, including improved locomotion and anxiolytic effects, of both protocols individually and in combination. The behavioral alterations occurred simultaneously with a positive impact on the BDNF/TrkB axis and a decrease in glial fibrillary acidic protein (GFAP) expression, as shown in Table 2B. Although they lacked a declarative statement comparing the effectiveness of the applied therapeutic procedures, the authors proposed that their impact might be partly related to their neuroprotective effect on attenuating astroglial activation and BDNF decrease. Another investigation into the impact of magnetic fields on depressive-like behavior in rats was conducted by Yang and colleagues [71]. The response was evaluated in the specific model of poststroke depression (induced by middle cerebral artery occlusion, followed by the CUMS procedure), using cerebellar intermittent theta burst stimulation (iTBS) with different intensities (1800, 2400, and 3000 pulses) and total time (720, 960, and 1200 s), in 60 cycles for two weeks. The applied protocols reversed specific patterns of poststroke depressive-like behavior (decreased vertical movement, locomotor distance, and sucrose preference, and increased immobility time and balance beam test score). In addition, a morphological evaluation of iTBS effects showed increased dendritic length and spine density in Purkinje cells and reduced neuronal damage in specific brain regions (Table 2B). Simultaneously, iTBS protocols produced increased BDNF levels, resulting in the activation of the cAMP/PKA/CREB pathway. Previously, the more frequently used method for induction of agitated depression in mice, olfactory bulbectomy, was applied by Heath and investigators [72] to explore the potential differences in the effect achieved by medium- and high-intensity rTMS. Both medium- and high-intensity rTMS (10 Hz, 3 min, 50 mT for medium intensity, or 1 T for high intensity) were administered five days per week for 4 weeks and diminished psychomotor agitation as analyzed in the forced swim test; however, only medium-intensity rTMS increased BDNF levels, cell proliferation, and neurogenesis in the hippocampus and prefrontal cortex (Table 2B). The authors concluded that the applied methods of rTMS induced different neurobiological alterations in a mouse model of agitated depression.

Ultimately, it becomes obvious that the majority of clinical trials and all preclinical investigations confirmed significant improvement following transcranial stimulation techniques according to clinical and/or behavioral criteria. In contrast, peripheral neurotrophin changes that occur concomitantly with rTMS-mediated therapeutic protocols do not consistently demonstrate reliable changes in peripheral BDNF or other neurotrophins, despite observed clinical improvement. This notable heterogeneity may be related to differences in stimulation parameters and sample characteristics, as well as assay methods, suggesting that peripheral neurotrophin changes should be considered exploratory rather than validated biomarkers in the context of transcranial stimulation techniques at this stage of investigations. In that sense, this is currently a developing issue in the field of rTMS implementation.

#### 3.1.2. Transcranial Current Stimulation

Since 2000, when Nitsche and Paulus [75] published a seminal study demonstrating that applying anodal transcranial direct current stimulation (tDCS) over the motor cortex increases cortical excitability, which is usually decreased in depressive patients, various modalities of transcranial electrical stimulation, including tDCS and transcranial alternating current stimulation (tACS), have been considered a promising non-invasive approach for treating depressive disorders. The principal advantage of this methodology lies in the potential for home-based application, which can improve accessibility for patients with mobility limitations. Numerous studies have evaluated the neurophysiological mechanisms involved in transcranial electrical brain stimulation, especially in the field of depression. Many researchers have examined the role of neurotrophin system elements in linking tDCS administration to its clinical outcomes. However, the results remain contradictory. Some inconsistencies could potentially be addressed through the refinement and standardization of protocols.

An initial trial conducted by Loo and colleagues [76] aimed to assess the role of BDNF following transcranial direct current stimulation (tDCS) in patients with unipolar and bipolar depression. The authors applied protocols with high (active, 2.5 mA, 30 min) and low current (0.034 mA and two 60-s current ramps up to 1 and 0.5 mA) tDCS to the left prefrontal cortex (20 sessions for 4 weeks) in both groups of patients. The extensive evaluation of clinical outcome performed using numerous scales (MADRS, CGI, and Quick Inventory of Depressive Symptomatology self-report) showed that mood improved significantly over the treatment period in both unipolar and bipolar groups. Interestingly, the antidepressant effect was also observed following the low-current protocol, although it was initially intended to be a positive control (Table 3). Moreover, the authors estimated the potential impact of BDNF polymorphism on the outcome of the applied protocols (without quantifying BDNF levels); however, no correlation was identified.

**Table 3:** An overview of transcranial current stimulation methods’ impact on the neurotrophin system in depression.

Applied Methodology	Diagnosis		Neurotrophin System Alterations			Clinical Outcome	Ref.
	Type	Specific Features	Neurotrophins	Neurotrophin Receptors	Downstream Mechanisms		
CLINICAL TRIALS							
tDCS—high current (2.5 mA, 30 min) and low current (0.034 mA, two 60 s current ramps up to 1 and 0.5 mA) in 20 sessions for 4 weeks	Unipolar and bipolar depression (*n* = 130)	+ paroxetine	-	-	-	Positive	[76]
tDCS (left DLPFC, 0.5 mA, for 10 days) as 30 and 20 min sessions	Moderate depression (*n* = 69)	+ sertraline	BDNF n.c.	-	-	Positive	[77]
tDCS (2 mA for 30 min, 22 sessions—15 in the first 3 weeks and 7 from week 3 to 10, once a week)	MDD (treatment-resistant, chronic, and recurrent) (*n* = 236)	+ escitalopram	BDNF n.c. GDNF n.c. NGF n.c.	-	Neuroinflammation ↓	Positive	[78]
tDCS (2 mA, 30 min/d, 10 days, and another 2 times, one at week 4 and at week 6)	Moderate to severe bipolar depression (*n* = 52)	-	BDNF n.c. GDNF n.c. NGF n.c.	-	Neuroinflammation ↓	Positive	[79]
ANIMAL MODEL							
tDCS (prefrontal, 50 μA anodal tDCS (13.33 A/m^2^) 15 min): acute (single) chronic (14 days)	Depression in adolescent rats	Olfactory bulbectomy	BDNF ↑	-	5-hydroxytryptamine downstream mechanisms ↑	Positive	[80]

Arrows (↑ or ↓) indicate *p* < 0.05; n.c. indicates *p* > 0.05; *n* indicates the number of subjects.

The clinical trial performed by Pavlova and researchers [77] on moderately depressed patients was focused only on tDCS effects, but in combination with the antidepressant drug sertraline. tDCS was applied (left DLPFC, 0.5 mA, for 10 days) as 30- and 20-min sessions. The clinical outcome, quantified using HDRS and BDIS, confirmed the benefits of tDCS (Table 3) when combined with sertraline in depression treatment (more convincing results were achieved with the 30-min protocol). Interestingly, the applied protocols did not affect BDNF levels. However, the experimental design of this study prevented the quantification of tDCS effects when used alone.

The study by Brunoni and coworkers [78] included a wide range of patients with treatment-resistant, chronic, and recurrent depression to evaluate the combined effects of tDCS (2 mA for 30 min; 22 sessions total: 15 sessions in the first 3 weeks and 7 sessions from week 3 to week 10, once a week) and escitalopram. The combined treatment protocol demonstrated an antidepressant effect, as measured by the HDRS-17, showing both early (after three weeks) and long-term (at the ten-week endpoint) responses, with patients reporting a significantly improved mood after ten weeks of treatment. The authors used various biomarker evaluations. While the plasma levels of some proinflammatory cytokines decreased in a timely manner, the elements of the neurotrophin system (BDNF, GDNF, and NGF) showed no significant alterations (Table 3). However, the authors suggested that NGF baseline levels may be used for predicting the DCS/escitalopram response in depressive patients.

In the study that followed, a research group led by Brunoni [81] showed that BDNF polymorphism did not significantly influence the effects of tDCS and escitalopram in MDD patients.

In contrast to the previously presented studies, Goerigk and investigators [79] performed tDCS (2 mA, 30 min daily for 10 days, followed by two additional sessions at week 4 and week 6) in patients with bipolar depression, without the co-administration of antidepressant drugs. The examined neurotrophic factors (BDNF, GDNF, and NGF) remained unchanged by the tDCS protocol (Table 3), whereas the cytokine profile exhibited significant alterations, thereby validating the anti-inflammatory response to tDCS. Moreover, the authors observed that IL-6 alterations (increased pretreatment values and significant decline in conjunction with tDCS) may be a predictive factor for successful clinical outcomes (as confirmed by HDRS-17).

Unlike studies that have explored the impact of TMS on neurotrophin system elements in MDD, there is insufficient data from animal experimental models to support the effects of tDCS (Table 3). Thus, the only preclinical study addressing this issue was conducted on adolescent male rats, and it included the animals with olfactory bulbectomy, as a model of depressive-like behavior induction, treated with tDCS (prefrontal, 50 μA anodal tDCS (13.33 A/m^2^) 15 min), both acute and chronic (14 days), and/or pharmacologically (paroxetine) [80]. The results indicated that the benefits of the current stimulation protocol were evident in the early phase (after two days) as a reduction in paroxetine-induced anhedonia. Additionally, chronic administration of tDCS over 14 days led to a decrease in agitated depressive-like behavior, as measured by the open field test, and reversed the decline in the sucrose preference test when combined with paroxetine. Simultaneously, co-treatment with tDCS and paroxetine normalized BDNF levels and 5-HT effects in various brain regions. Based on the study’s findings, the authors suggested that using tDCS alongside antidepressant medication could serve as an adjunctive treatment. This approach may help prevent the adverse effects associated with paroxetine and improve its long-term therapeutic benefits.

Although several clinical studies conducted over the last decade have demonstrated the efficacy of tDCS/tACS, by means of clinical improvement, current evidence does not show consistent changes in neurotrophin system elements. This suggests that this underlying mechanism remains inadequately characterized and insufficiently investigated in the causal context of clinical trial design, with a notable deficiency in preclinical investigations. Obviously, more research is needed to determine the role of the neurotrophin system in the beneficial effects of transcranial current stimulation techniques, but with more standardized protocols in clinical trials, and adjusted protocols in preclinical experimental procedures that would allow for a translational approach.

#### 3.1.3. Electroconvulsive Therapy

Almost a century ago, ECT was introduced, while the initial results of clinical trials evaluating the clinical outcome of this groundbreaking method for severe psychiatric conditions, predominantly a spectrum of depressive disorders, were presented in the early 1940s of the 20th century [82]. Since then, numerous modifications to this method have been made, along with attempts at standardization and adjustments to ethical principles, which have also evolved. However, in present-day clinical practice, ECT is still considered a highly effective treatment for TRD. Even more, it is considered a first-line treatment for severely depressed patients with life-threatening inanition, when a fast response is substantial due to a high suicide or homicide risk, as well as in the conditions of extreme agitation, psychosis, or stupor [83]. A plethora of evidence for the neurobiological mechanisms underlying ECT antidepressant effects has been published over the decades. However, there is insufficient data emphasizing pathways that involve neurotrophin system elements in ECT therapeutic action.

The data obtained over the last decade clearly indicate that most studies conducted five years ago and earlier did not confirm the significance of neurotrophins in mediating the clinical benefits of ECT application. In contrast, more recent publications consistently highlight the role of neurotrophins, particularly BDNF, and even suggest their use as biological markers for predicting clinical outcomes. The observed difference appears to coincide with methodology standardization, i.e., the predominant placement of bitemporal electrodes in recent clinical trials and certain improvements in assay methodology.

Ryan and investigators [84] performed the ECT protocol with hand-held electrodes twice a week, the mean number being eight sessions per patient, with a moderate-dose bitemporal (1.5 × seizure threshold) and a high-dose unilateral (6 × seizure threshold) to test the response in BDNF blood levels to ECT. Confirming the evident BDNF gene polymorphism in the patient’s group, there is still no association between plasma BDNF levels and depression severity or the clinical response to ECT. Table 4A shows that Sorri and collaborators [85] reported no data supporting a connection between blood BDNF levels and the clinical outcome of ECT (bilateral electrode placement, three times a week) in MDD. Bouckaert and researchers [86] also reported a lack of connection between plasma BDNF levels and the clinical outcome of ECT. Applying a similar ECT protocol (unilateral, twice a week, according to Dutch guidelines [87], the authors presented results with no change in BDNF levels in MDD late-life patients during the twelve sessions, as well as in the follow-up. At the same time, the applied protocol resulted in increased hippocampal volume that correlated with the clinical outcome, but without the expected involvement of the estimated neurotrophic factor.

**Table 4:** (A). An overview of electroconvulsive stimulation methods’ impact on the neurotrophin system in MDD patients. (B). An overview of electroconvulsive stimulation methods’ impact on the neurotrophin system in depression-induced animal models.

**(A)**							
**Applied Methodology**	**Diagnosis**		**Neurotrophin System Alterations**			**Clinical Outcome**	**Ref.**
	**Type**	**Specific Features**	**Neurotrophins**	**Neurotrophin Receptors**	**Downstream Mechanisms**		
ECT (hand-held electrodes)	MDD (*n* = 111)		BDNF n.c.	-	-	Positive	[84]
ECT (bilateral electrode placement)	MDD (*n* = 30)		BDNF n.c.	-	-	Positive	[85]
ECT (unilateral electrode position)	MDD (*n* = 88)	Late-life patients	BDNF n.c.	-	Hippocampal volume ↑	Positive	[86]
ECT (unilateral/bilateral electrode position)	MDD (*n* = 61)	Late-life patients	BDNF n.c.	-	Hippocampal volume ↑	Positive	[88]
ECT (bilateral electrode position)	MDD (*n* = 35)		BDNF n.c.	-	-	Positive	[89]
ECT (unilateral electrode position)	MDD (*n* = 31)	Unipolar and bipolar	BDNF ↑ (when low baseline)	-	-	Positive	[90]
ECT	MDD (*n* = 94)		BDNF n.c.	-	-	Positive	[91]
ECT (bilateral electrode position)	MDD (*n* = 74)		BDNF n.c.	-	-	Positive	[92]
ECT (unilateral electrode position)	MDD (*n* = 24)		pBDNF n.c. sBDNF ↑	-	-	Positive	[93]
ECT (unilateral electrode position)	MDD (*n* = 13)		sBDNF ↑	-	-	Positive	[94]
ECT (unilateral/bilateral electrode position)	MDD (*n* = 9)		BDNF (in CSF) ↑ sBDNF n.c.	-	-	Positive	[95]
ECT (unilateral/bilateral electrode position)	MDD (*n* = 36)		sBDNF ↑	-	-	Positive	[96]
ECT	MDD (*n* = 60)	+ aerobic exercise	BDNF ↑	-	-	Positive	[97]
ECT (unilateral electrode position)	MDD (*n* = 99)	Late-life patients	BDNF ↑ (n.s.)	-	Neuroinflammation ↓ TNF-α/BDNF ratio ↓	Positive	[98]
ECT (bitemporal electrode position)	MDD (*n* = 19)	Unipolar and bipolar	BDNF ↑	-	BDNF/ERK1/CREB ↑	Positive	[99]
ECT (bitemporal electrode position)	MDD (*n* = 75)		BDNF ↑	-	Neurogenesis ↑	Positive	[100]
ECT (bitemporal electrode position)	MDD (*n* = 9)		BDNF ↑	-	Neuroinflammation ↓	Positive	[101]
Arrows (↑ or ↓) indicate *p* < 0.05; n.c. indicates *p* > 0.05; *n* indicates the number of subjects.							
**(B)**							
ECS (corneal electrode, 100 pulse/s, 0.3 ms, 1 s shock, and 50 mA)	CUMS—adult mice		BDNF ↑	-	Neurogenesis ↑	Positive	[102]
ECS	CUMS—rats		BDNF ↑	-	Ferroptosis ↓	Positive	[103]
ECS (7 sessions—100 pulse/s, 3 ms, 1 s shock duration, 50 mA, for 15 days)	BDNF disrupts production—mice	Bdnf-e1 mice	-	-	Neuroplasticity ↑	Positive	[104]
ECS (ear clip electrodes 55 to 70 mA in 0.5 s, 100 Hz/d, for 10 days)	Flinders Sensitive and Resistant Line rats	Prone to depressive-like behavior	BDNF ↑	-	Neurogenesis ↑ Neuroplasticity ↑	Positive	[105]
ECS (40 mA on the first 5 days and 50 mA on the following 5 days for 0.5 s, 50 Hz)	Rats	Naive	pro-BDNF n.c. BDNF ↑	-	BDNF/TrkB ↑	-	[106]
Arrows (↑ or ↓) indicate *p* < 0.05.							

This is in line with the results obtained in the study conducted by Van Den Bossche’s team [88], which also indicated that ECT-induced hippocampal volume increase did not involve BDNF-dependent mechanisms. Accordingly, one of the pioneer studies [89], despite recognizing the significant difference in the baseline serum levels of BDNF, did not observe any difference in BDNF levels following the ECT protocol (twice-weekly brief-pulse bitemporal ECT was administered using hand-held electrodes). This finding is consistent with the data presented by Freire and colleagues [107] in the study, which demonstrated that serum BDNF levels did not change when ECT was combined with antidepressant drugs.

Furthermore, Freire and colleagues [90] evaluated the impact of the ECT protocol (three times a week, unilateral electrode placement, according to previously described guidelines [108] on clinical outcomes in MDD patients (unipolar and bipolar) and its correlation to BDNF level alterations. According to the results obtained in this study, it appears that subjects with lower baseline BDNF levels are more likely to respond positively (increase) to ECT in contrast to those with higher baseline BDNF levels, who showed no significant change following ECT. Furthermore, the clinical outcome was significantly better in the low-level baseline group of MDD patients (Table 4A). The authors concluded that initial BDNF values might be considered predictive factors for remission. In contrast, the clinical trial conducted by van Zutphen and collaborators [91] did not show confirmation that baseline BDNF levels could be considered an eligible biomarker for ECT outcomes in clinical practice following the ECT protocol (according to [109]). Moreover, the trial conducted simultaneously by Maffioletti and her team [92] led to the same conclusion, based on MADRS scoring one month after the completion of the ECT protocol. Once again, Stephani and colleagues [110] expressed skepticism about the role of BDNF in mediating the antidepressant effects of ECT. Their trial, which involved at least 12 consecutive ECT sessions, found no significant influence of BDNF polymorphism in patients with MDD.

An intriguing experimental design employed by Vanicek and collaborators [93] enabled frequent monitoring that includes an early response to ECT, in accordance with ECT guidelines [111] through the measurement of BDNF blood levels in both serum and plasma. Although the authors reiterated their previous finding of no association between serum BDNF levels and the clinical response to ECT [84], the results indicated that serum BDNF levels significantly increased throughout the protocol (Table 4A). This increase began with the initial session and peaked one month after the last ECT session, showing an interesting boost after each session. In contrast, this pattern was not observed for plasma BDNF levels. The continuation of ECT conducted by the same research team [94] confirmed the relationship between the ECT-induced increase in serum BDNF concentrations and remission status. This suggests that a stable clinical condition was maintained throughout the continuation of the ECT protocol. To address the contradictory results regarding the presence or absence of BDNF response to ECT, it is important to note that a preliminary report by Mindt and investigators [95] showed that BDNF levels significantly increase in cerebrospinal fluid following approximately 12 sessions of ECT (three per week) before any significant change occurs in peripheral blood. Another attempt to use BDNF alterations as a predictive factor for ECT clinical outcomes was proposed by Kranaster and coworkers [96]. The authors examined the seizure quality index, noting a positive correlation with serum BDNF levels following individual ECT sessions. They suggested that the relationship between seizure quality and the antidepressant response to ECT may depend on BDNF levels.

Salehi and investigators [97] implemented two protocols that were anticipated to induce antidepressant effects in MDD patients: aerobic exercise and ECT, both of which were implemented individually and in conjunction. Both protocols applied, ECT (three times a week for four consecutive weeks) and aerobic exercise (three sessions per week lasting 40–45 min/per session on a treadmill), resulted in antidepressant effects while also increasing plasma BDNF levels (see Table 4A). The combined group achieved the best results for both estimated parameters. The impact of the other ECT protocol was also evaluated in patients with late-life MDD [98]. The protocol was administered twice a week over approximately 12 sessions. This duration depended on whether patients achieved remission or exhibited no further clinical progress during the previous two weeks of ECT sessions, with a minimum of six treatments required. The MADRS score showed a significant decline over time, beginning three weeks after treatment commenced and continuing until one week following the final ECT session. At the same time, blood samples were collected, and the analysis showed a slight increase in BDNF levels and a decrease in TNF-α and IL-6 levels (Table 4A). To establish the connection between the status of the neurotrophin system and neuroinflammatory markers as part of the pathophysiological background of MDD, the authors indicated that there was a significant negative correlation between the TNF-α/BDNF ratio and the clinical manifestations of depression.

More detailed information considering the impact of ECT on the neurotrophin system was presented in the results of a clinical trial performed by Schurgers and colleagues [99]. ECT was administered twice a week, following the protocol established by Espinoza and Kellner [112] to patients with both unipolar and bipolar MDD. Each patient received an average of six sessions, with treatment ceasing when no improvement was observed or when patients reached remission. After completing ECT protocols, the patients showed significant improvement, according to the HDRS and BDI scales, which was not pronounced in follow-up. Blood sample analysis revealed increased expression of BDNF (Table 4A), as well as indicators of its downstream mechanisms’ activity (ERK1 and CREB). Further analysis confirmed that elements of the BDNF/ERK1/CREB pathway showed the best correlation with clinical outcome measures.

Xie and the team [100] presented the first study to provide human in vivo evidence of early neurogenesis in treatment-resistant MDD patients undergoing ECT and to investigate the potential role of BDNF. ECT protocols [113] were performed three times a week for up to twelve sessions. ECT protocols showed beneficial effects by means of clinical outcomes while simultaneously increasing plasma BDNF levels (Table 4A). Isolating neuron-derived extracellular vesicles before and after the last ECT session gave the authors perspective on the markers of neurogenesis (doublecortin and cluster of differentiation 81). More specifically, the levels of doublecortin were lowered in MDD patients at baseline and significantly increased in response to ECT, while CD81 was found to be higher in MDD patients at baseline but did not change after the ECT treatments. This original approach clearly demonstrated the benefits of ECT in promoting previously declined neurogenesis in treatment-resistant MDD patients.

This is in line with the most recent findings obtained by Falhani and collaborators [101], who showed that ECT protocol resulted in a significant increase in serum BDNF levels concomitantly with the downregulation of the proinflammatory marker (IL-4) after the end of the ECT series (3 ECT sessions per week for 6 weeks), which may be associated with clinical improvement (quantified by the HAMD-21 and BDI scales).

The number of preclinical studies examining the impact of electroconvulsive stimulation (ECS) on depressive-like behavior, particularly regarding the roles of neurotrophin system elements, is not as impressive as that of clinical trials. A chronic stress-induced depression model in adult mice was used by Maynard and colleagues [102] to assess the effectiveness of ECS. Chronic stress-induced corticosterone-mediated depression was manifested by dendrite atrophy and BDNF deficiency in the cingulate cortex (Table 4B); however, this condition was reversed by the applied ECS protocol (corneal electrode, 100 pulse/s, 0.3 ms, 1 s shock, and 50 mA). The other model designed to promote corticosterone-mediated expression was developed by Kobayashi and Segi-Nishida [114]. They utilized an indirect approach involving long-term exposure to adrenocorticotropic hormone to assess the impact of ECS on depressive-like behavior in mice. However, this methodology failed to confirm a reliable depression model, so all additional results should be considered invalid.

To evaluate the effects of ECT in an animal experimental model, Li and team members [103] used the method of CUMS-induced depression in rats. ECT protocol (10 days) resulted in an antidepressant effect that was accompanied by increased numbers of hippocampal neurons, increased expression of negative regulators of ferroptosis (including glutathione peroxidase 4, ferritin heavy chain 1, and ferroptosis suppressor protein 1), upregulation of BDNF and nuclear factor erythroid 2-related factor, and downregulation of acyl-CoA synthetase long-chain family member 4 (a positive regulator of ferroptosis) in the hippocampus. Those beneficial effects were potentiated by the simultaneous administration of etomidate, which enhanced the antidepressant effect of ECT by protecting hippocampal neurons against ferroptosis.

In their quest to identify an additional mechanism linking BDNF alterations to its beneficial role in the ECS-induced antidepressant effect, Ramnauth and collaborators [104] investigated using Bdnf-e1 mice, which are genetically engineered to disrupt BDNF production from promoter I. The implementation of viral vectors to compensate for specific knockout deficiency revealed that loss of promoter I-derived BDNF caused changes in spine density and morphology in BDNF exon 1-expressing neurons following ECS (7 sessions with shock parameters: 100 pulse/s frequency, 3 ms pulse width, 1 s shock duration, and 50 mA current, for 15 days). Based on the obtained data, Bdnf exon 1-expressing neurons in the mouse piriform cortex appear to play a crucial role in enhancing structural plasticity within the neurons of the piriform cortex. This enhancement may represent one of the mechanisms underlying the antidepressant effects of ECS.

Another modification predicting an increased pro-depressant phenotype was utilized by Chen and colleagues [105] through genetically driven phenotype modeling for clinical depression. They specifically employed the Flinders Sensitive and Resistant Line rats to evaluate alterations accompanied by ECS (ear clip electrodes using 55 to 70 mA in 0.5 s at a frequency of 100 Hz square wave pulses, daily for 10 days) that may involve a BDNF-driven mechanism. ECS treatment increased hippocampal BDNF expression levels, which was accompanied by several morphological alterations, such as the increased volume of the hippocampal CA1 region, the increased number of synapses, and the rise in the number and volume of mitochondria (Table 4B). The antidepressant effect of ECS was confirmed in the forced swim test, and the authors proposed that ECS is a rapid and efficient therapeutic method for treating depressive-like behavior, potentially linked to BDNF increases related to downstream mechanisms involved in neurogenesis. The team of investigators led by Chen [115] confirmed the results from the pivotal study by analyzing the late follow-up, which occurred three months after the last ECT session. They presented evidence of sustained ultrastructural changes in the hippocampus.

The study conducted by Enomoto and colleagues [106] warrants special attention, as the authors examined the receptors and downstream mechanisms while analyzing the effects of ECS (40 mA for the first 5 days and 50 mA for the subsequent 5 days, delivered for 0.5 s at 50 Hz) treatment in the rat hippocampus. The study, although conducted on healthy animals, revealed that the ECS procedure led to an increase in BDNF expression in the ventral hippocampus. This increase was accompanied by elevated phosphorylated TrkB expression in both the dorsal and ventral hippocampus, resulting in enhanced BDNF/TrkB signaling following multiple ECS treatments (Table 4B). The authors reported an upregulation of mature BDNF in the examined hippocampal regions, while the expression of its precursor, pro-BDNF, remained unchanged.

Some more recently introduced methods for non-invasive transcranial brain stimulation (transcranial random noise stimulation, transcranial photobiomodulation, transcranial ultrasound stimulation, and trigeminal nerve stimulation) have also been evaluated for the potential evidence of neurotrophin system elements involved in their antidepressant action. However, the current number of studies is still insufficient to result in reliable conclusions on this issue.

#### 3.1.4. Transcranial Photobiomodulation

Photobiomodulation (PBM) is a form of light therapy that involves the application of red or near-infrared light, which modulates physiological processes by enhancing cerebral blood flow, reducing inflammation, inhibiting apoptosis, and promoting neurogenesis [116]. Starting with Lewy and colleagues’ initial case report that bright artificial light treatment may be beneficial for manic-depressive patients with a seasonal mood cycle [117], light therapy has been proposed for the treatment of neuropsychiatric disorders, including MDD.

The investigators in this field, unfortunately, did not accept a very promising approach to addressing depressive-like behavior, such as the model of transcranial photobiomodulation. Despite its complete invulnerability, only the team led by Farazi [118] examined the effects of tPBM (810 nm wavelength, 8 J/cm^2^ fluence, 10 Hz pulsed wave mode) for two weeks and an enriched environment (EE) (either independently or in conjunction) on a mouse model of noise-induced stress (for the induction of depression). Both tPBM and EE protocols, as well as their combinative form, resulted in an antidepressant effect (confirmed in the forced swimming test), concomitantly with an anxiolytic effect (open field and elevated plus maze test). The benefits observed in behavioral testing were accompanied by a significant increase in the hippocampal BDNF/TrkB/CREB expression (Table 5). The authors proposed that the improvement in behavioral outcomes could be due to increased activity in the BDNF/TrkB/CREB signaling pathway in the hippocampus.

**Table 5:** An overview of other non-invasive transcranial stimulation methods’ impact on the neurotrophin system in depression.

Applied Methodology	Diagnosis		Neurotrophin System Alterations			Outcome	Ref.
	Type	Specific Features	Neurotrophins	Neurotrophin Receptors	Downstream Mechanisms		
tPBM (810 nm, 8 J/cm^2^ fluence, 10 Hz pulsed wave mode) for 2 weeks	Noise-induced depression in mice	+ enriched environment	BDNF ↑	TrkB ↑	BDNF/TrkB/CREB ↑	Positive	[118]
TUS (15 min stimulation of the prelimbic cortex every day) for 2 weeks	Restraint stress-induced depression in rats		BDNF ↑	-	-	Positive	[119]
TUS (dorsal lateral nucleus 30 min/d) for 3 weeks	Corticosterone-induced depression in mice		BDNF n.c.	-	5-hydroxytryptamine ↑	Positive	[120]

Arrows (↑) indicate *p* < 0.05; n.c. indicates *p* > 0.05.

#### 3.1.5. Transcranial Ultrasound Stimulation

In the search for treatments for drug-resistant depression that rely on non-pharmacological methods, new neurostimulation techniques, such as transcranial ultrasound stimulation (TUS), have been proposed. These techniques are considered noninvasive and precise, allowing for targeted neuromodulation of deep brain regions. However, given concerns about clinical translation of TUS (questionable precision, safety, and portability), the only evidence for neurotrophin system involvement in MDD patients’ response to this technique remains limited to recent preclinical investigations. Zhang and investigators [119] explored the impact of TUS on the depression rat model induced by the restraint method. TUS (15 min of stimulation of the prelimbic cortex every day for 2 weeks) successfully produced antidepressant-like effects in behavioral analysis (sucrose preference test), which was accompanied by an increase in hippocampal BDNF expression (Table 5). The authors suggest that low-intensity TUS might be a promising therapeutic strategy for depression as an adjunctive therapy. Wu and collaborators [120] examined the effects of TUS applied to the dorsal lateral nucleus for 30 min daily over a period of three weeks in a mouse model of depression induced by corticosterone (20 mg/kg for three weeks). TUS significantly improved depression-like behaviors, as measured by the sucrose preference and tail suspension tests, and it also reduced anxiety-like behaviors in the elevated plus maze test. The TUS application resulted in a non-significant increase in the 5-HT levels and a significant decrease in the noradrenaline levels, but it did not affect the levels of DA and BDNF. Although most authors are suspicious of this kind of adjuvant therapy, due to its safety, it should be considered as a potential technique for remedying depression and anxiety comorbidity.

### 3.2. An Overview of Invasive Methods of Transcranial Stimulation Impact on the Neurotrophin System Elements in the Treatment of MDD

Not surprisingly, the number of studies evaluating invasive transcranial stimulation techniques for treating MDD, which included those examining changes in neurotrophin system elements, is notably limited. Furthermore, there is a clear lack of data from clinical trials, which forces us to depend on results from preclinical investigations that utilize methodologies similar to those used in deep-brain stimulation (DBS), vagus nerve stimulation (VNS), and epidural cortical stimulation.

#### 3.2.1. Deep Brain Stimulation

Although initially employed in the treatment of patients with neurodegenerative disease [121], the pioneering work of Mayberg and his team revealed the benefits of the deep brain stimulation technique in treatment-resistant depressive patients [122]. Liu and colleagues [123] performed an investigation to test a preclinical paradigm for MDD treatment using bilateral ventromedial prefrontal cortex (vmPFC) DBS in the rat model of chronic unpredictable stress. After 28 days of depressive-like behavior, the authors applied DBS (130 Hz, 200 μA, 90 μs pulses administered 5 h daily for 7 days) by implementing electrodes stereotaxically in vmPFC. The applied protocol, as presented in Table 6, was sufficient to reverse depressive-like behavior in rats, according to the results obtained in the battery of behavioral tests (sucrose preference test, open field test, elevated plus maze test, and forced swim test), simultaneously with the enhancement of the BDNF/TrkB signaling pathway accompanied with its downstream ERK1/2 activity, which was not observed in non-stressed rats. This methodological approach confirmed that this type of intervention on the vmPFC, as a promising target region, may lead to a beneficial impact on the molecular basis of MDD. After seven weeks of the CUMS procedure in rats, Sun and coworkers [124] employed the unilateral DBS of vmPFC (20 Hz, 1 h daily) for 28 days to estimate its potential antidepressant effect. The series of behavioral tests (sucrose preference, open field, and forced swim test) confirmed the welfare of this methodology in amelioration of depressive-like behavior, but also in reversing cognitive impairment (Morris water maze). The behavioral improvement occurred simultaneously with increased hippocampal expression of BDNF and activity of the BDNF/mTOR signaling pathway, which restored the number of synapses (Table 6).

An earlier investigation conducted by Dandekar and his team [125] explored the potential therapeutic effects of DBS (130 Hz, 200 μA amplitude, 90 μs pulse width, 8 h/day for one week) when applied unilaterally to the middle forebrain bundle following a CUMS protocol for 42 days and provided evidence on the specific neuroprotective mechanisms of this methodology. A modification of the anhedonia test (Froot Loops Consumption Assay) [130], as shown in Table 6, indicated a reduction in depressive-like behavior. This change occurred together with the increase in hippocampal BDNF levels, which reversed the decline induced by the previous CUMS treatment. Moreover, both pro- and antidepressant protocols applied in this study significantly influenced the neuroinflammatory profile in the obtained samples (increased levels of IL-1β, IL-2, IL-5, IL-6, IL-7, IL-18, TNF-α, and INF-γ in CUMS rats, with modulation following DBS).

One of the pioneering investigations, considering the impact of DBS on neurotrophin system elements in the treatment of depressive states, was conducted by Jiménez-Sánchez and collaborators [126]. A non-specific method (olfactory bulbectomy-OBX) was used for the induction of behavioral alterations in rats, including depressive-like behavior induction, and its efficiency was confirmed by sucrose-preference and forced swimming tests. Acute DBS was applied in the form of bipolar stimulation with electrodes implanted bilaterally in the infralimbic medial prefrontal cortex, IlmPFC (130 Hz, 200 μA, 90 μs, for 1 h), and resulted in a significant antidepressant effect. This effect was accompanied by increased levels of BDNF, as well as ERK/CREB and BDNF/mTOR signaling. The authors indicated that the methodology used aligns with the antidepressant responses observed with other rapid-acting antidepressant drugs (Table 6).

#### 3.2.2. Vagus Nerve Stimulation

Since its initial use for controlling epileptic seizures in dogs [131], vagus nerve stimulation has been employed in treating a variety of mental diseases, including treatment-resistant depression [132]. However, there are still concerns about its therapeutic use. Nevertheless, the only data indicating the involvement of neurotrophin system elements in VNS action in the depressive state were obtained through preclinical research.

The chronic restraint stress (CRS) protocol was conducted by Shin and colleagues [127] over a two-week period to induce a prodepressant response, allowing for an evaluation of the effects of VNS. The chronic procedure, which involved an implanted electrode set to 10 mA, 5 Hz, and a pulse duration of 5 ms for 5 min over 14 days, resulted in significant antidepressant action in the rats that exhibited increased depressive levels, as confirmed by forced swimming and open field tests. This effect was also accompanied by an increase in BDNF expression levels in the hippocampus (Table 6). It is intriguing to observe that the administration of BDNF alone did not result in any meaningful behavioral changes. This suggests that the more extensive impact of VNS may be more significant, as VNS-mediated serotonergic input via 5-HT1B receptors into the hippocampal neurons may more accurately activate BDNF pathways (p-Erk1/2 activity) and attenuate depressive-like behaviors in CRS rats.

Unlike previous investigations that relied on established prodepressant protocols, the recent study by Valente and colleagues [128] offers a unique perspective on this methodology as it is applied to intact subjects, specifically healthy rats. The experimental design was based on the estimation of acupuncture stimulation of the auricular branch of the vagus nerve (ABVN) for the evaluation of various behavioral aspects, including memory, anxiety-like, and depression-like behaviors. The beneficial impact of this invasive methodology (acupuncture stimulation using needles implanted 1–2 mm from the surface of the pinna skin, initially rotated and then replaced with fixed needles) on depressive states was not confirmed by the sucrose preference and forced swimming tests, although the anxiolytic effect was maintained. Moreover, the authors noted a significant rise in hippocampal BDNF levels (Table 6), suggesting that the absence of antidepressant effects following the implemented methodology may be due to an inadequate number of experimental design tasks, such as animal preconditioning, and the interval between protocol completion and behavioral testing.

Furthermore, Shah et al. [129] made an excellent attempt to compare the conventional (pharmacological) approach to the alternative (VNS) approach by investigating the effects of desipramine and VNS. The effects of desipramine were analyzed following acute (15 mg/kg s.c.) and chronic (10 mg/kg/day via osmotic pump i.p. for 24 days) administration, while VNS acute treatment was implemented according to the Furmaga protocol (current 0.25 mA, frequency 20 Hz, pulse width 0.25 ms, duty cycle of 30 s on, 300 s off) [133], and chronic VNS treatment (following the same stimulation parameters) was applied for 24 continuous days. Both the acute and chronic protocols demonstrated antidepressant effects in the forced swimming test; however, the chronic treatments resulted in significantly greater benefits in terms of behavioral improvement and increased expression of TrkB in the hippocampus (Table 6). Overall, the results of this study suggest that there is no significant difference in the quantification of benefits achieved through the two different methodological approaches.

It is evident that efforts to incorporate invasive transcranial stimulation methods into the treatment of MDD are fewer than those to integrate non-invasive methods, as indicated by published data from the past decade. Furthermore, there is an obvious lack of standardization in experimental design and protocols, leading us to conclude that we are still quite distant from achieving the potential for translational capability.

## 4. Conclusions

Summarizing the data collected over the last decade from assessments of the neurobiological mechanisms influencing the clinical outcomes of transcranial stimulation methods, especially those involving neurotrophin-system-mediated pathways, it is evident that there is an increasing interest in this topic. Obviously, at this stage, we cannot declare that findings are ready for clinical application, nor specify optimal protocols based on current evidence. There is still a significant need for improvement to enable the practical application of this data. To accomplish this, future investigations should incorporate certain adaptations, such as standardizing methodologies in both clinical trials and animal experimental models, as well as refining patient and/or species selection, which can direct us in defining patient populations who are most likely to benefit from these techniques. Furthermore, it will provide a guideline for choosing the preferred transcranial stimulation method (instead of using a random approach), while taking into consideration differences between patient groups. Similar interventions, by means of protocol standardization and adjustment for clinical trials, should be implemented in preclinical investigations. These changes will allow for a more successful translational approach. Furthermore, advanced imaging and molecular techniques should be employed to unravel the precise mechanisms linking neurostimulation to neurotrophin dynamics. Until then, neurotrophin system alterations should be considered exploratory rather than validated biomarkers in the context of transcranial stimulation techniques.

## 5. Future Directions

Comprehending the mechanisms associated with neurotrophins may improve neurostimulation protocols, identify potential biomarkers for therapeutic response evaluation, and facilitate the development of individualized neuromodulatory therapies. Bridging the gap between clinical application and neurobiological understanding would not only enhance the therapeutic potential of brain stimulation in MDD, but it may also contribute to a more comprehensive, mechanism-driven framework for future psychiatric interventions. To bridge the gap between the clinically reported beneficial outcomes and our understanding of the underlying mechanisms involving the neurotrophin system, simultaneous interventions in both clinical (such as unifying methodology and rigorous patient selection) and preclinical (by properly simulating a clinical setup for a translational approach) research are necessary, which could result in more accurate guidelines based on clinical outcomes and corresponding neurochemical markers.

This neurobiological perspective approach strengthens the rationale for using transcranial stimulation techniques and provides new opportunities for developing personalized and mechanism-based treatment strategies for depression. As our understanding of the interplay between neural stimulation and molecular pathways deepens, transcranial stimulation methods may evolve from a symptom-targeting approach to a true disease-modifying therapy.

## Figures and Tables

**Table 1:** Classification of transcranial stimulation methods (with the principal effects) for MDD treatment.

Non-Invasive		Invasive	
Method	Effect	Method	Effect
Transcranial magnetic stimulation (TMS)	- increased synaptic plasticity - membrane potentials modulation - network activation	Deep brain stimulation (DBS)	- neurotransmitter modulation - network activation - neuroprotection - neurogenesis
Transcranial direct and alternating current stimulation (tDCS, tACS)	- membrane potentials modulation - neurotransmitter modulation - oscillations synchronization	Vagus nerve stimulation (VNS)	- neurotransmitter modulation - increased neuroplasticity - anti-inflammatory and anti-apoptotic action
Transcranial random noise stimulation (tRNS)	- neural excitability modulation - neurotransmitter modulation	Epidural cortical stimulation	- increased synaptic plasticity - network modulation - increased release of endogenous opioids
Transcranial photobiomodulation (tPBM)	- mitochondrial function improvement - antioxidant and anti-inflammatory action - neurogenesis promotion		
Transcranial ultrasound stimulation (TUS) and focused ultrasound stimulation (FUS)	- vascular function modulation - neuronal activity modulation - oscillations modulation		
Trigeminal nerve stimulation (TNS)	- neurotransmitter modulation - vascular function modulation - anti-inflammatory action - brain metabolism modulation		
Electroconvulsive therapy (ECT)	- neurotransmitter modulation - network activation - increased synaptic plasticity - anti-inflammatory action		

**Table 6:** An overview of invasive transcranial stimulation methods’ impact on the neurotrophin system in depression.

Applied Methodology		Induced Depression	Neurotrophin System Alterations			Outcome	Ref.
Name	Protocol		Neurotrophins	Neurotrophin Receptors	Downstream Mechanisms		
DBS (vmPFC, bilateral)	130 Hz, 200 μA, 90 μs pulses, 5 h/d, 7 d	CUMS in rat experimental model	BDNF ↑	TrkB ↑	ERK1/2 ↑	Depressive-like behavior ↓	[123]
DBS (vmPFC, unilateral)	20 Hz, 1 h/d, 28 d	CUMS in rat experimental model	BDNF ↑	-	BDNF/mTOR ↑	Depressive-like behavior ↓	[124]
DBS (MFB, unilateral)	130 Hz, 200-μA amplitude, 90-μs pulse width, 8 h/d, 7 d	CUMS in rat experimental model	BDNF ↑	-	Neuroinflammation ↓	Depressive-like behavior ↓	[125]
DBS (IlmPFC, unilateral)	130 Hz, 200 μA, 90 μs, for 1 h	OBX in rat experimental model	BDNF ↑	-	ERK/CREB ↑ BDNF/mTOR ↑	Depressive-like behavior ↓	[126]
VNS (bipolar hook electrode, neck region)	10 mA, 5 Hz, 5 ms of pulse duration, 5 min, 14 d	CRS in rat experimental model	BDNF ↑	-	p-Erk1/2 ↑	Depressive-like behavior ↓	[127]
VNS (ABVN)	Acupuncture needles, pinna skin	None (healthy rats)	BDNF ↑	-	-	Depressive-like behavior n.c.	[128]
VNS (unilateral cervical region)	Acute: 0.25 mA, 20 Hz, 0.25 ms, 30 s on, 300 s off Chronic: 24 d	None (healthy rats)	-	TrkB ↑		Depressive-like behavior ↓	[129]

Arrows (↑ or ↓) indicate *p* < 0.05.

## Data Availability

No new data were created or analyzed in this study. Data sharing is not applicable to this article.

## Abbreviations

The following abbreviations are used in this manuscript:

MDD	Major Depressive Disorder
TMS	Transcranial Magnetic Stimulation
tDCS	transcranial Direct Current Stimulation
tRNS	transcranial Random Noise Stimulation
tPBM	transcranial Photobiomodulation
TUS	Transcranial Ultrasound Stimulation
FUS	Focused Ultrasound Stimulation
TNS	Trigeminal Nerve Stimulation
ECT	Electroconvulsive Therapy
ECS	Electroconvulsive Stimulation
DBS	Deep Brain Stimulation
VNS	Vagus Nerve Stimulation
BDNF	Brain-Derived Neurotrophic Factor
NT-3	Neurotrophin-3
NGF	Nerve Growth Factor
MADRS	Montgomery–Asberg Depression Rating Scale
CGI	Clinical Global Impression Scale
HDRS	Hamilton Depression Rating Scale
PFC	prefrontal cortex
CUMS	Chronic Unpredictable Mild Stress
TRD	Treatment-Resistant Depression
